# Detecting Aquatic Pollution Using Histological Investigations of the Gills, Liver, Kidney, and Muscles of *Oreochromis niloticus*

**DOI:** 10.3390/toxics10100564

**Published:** 2022-09-26

**Authors:** Sana Shahid, Tayyaba Sultana, Salma Sultana, Bilal Hussain, Khalid Abdullah Al-Ghanim, Fahad Al-Bashir, Mian Nadeem Riaz, Shahid Mahboob

**Affiliations:** 1 Department of Zoology, Government College University, Faisalabad 38000, Pakistan; 2 Department of Zoology, College of Science, King Saud University, P.O. Box 2455, Riyadh 11451, Saudi Arabia; 3 Texas A&M University, College Station, TX 77843, USA

**Keywords:** tilapia, histopathology, downstream, upstream, Haematoxylin, eosin

## Abstract

The present study aimed to determine the degree of changes in the histological architecture of the liver, gills, kidneys, and muscles of fish *Oreochromis niloticus* collected from different polluted river sites. Fish samples collected from the Faisalabad Fish Hatchery and upstream of Chakbandi drain acted as a control. Necrosis, hemorrhage, and epithelial hyperplasia were observed in the gills of fish inhabiting the river downstream of the Chakbandi drain entrance. Liver tissues were found to be affected by vacuolated cytoplasm, bile duct proliferation, melanomacrophages, and necrosis. In kidney tissues, shrinkage of the renal cortex, necrosis, and destructive renal tubules were observed. Histopathology of muscles indicates the presence of hypertrophy and swollen myofibers. In contrast, upstream specimens of fish exhibited mild tissue alterations. Histopathology of gills tissue showed vacuolization. Liver tissues indicated the presence of hypertrophy and more frequent Kupffer cells than usual. The vacuolation was also observed in kidney tissues. Muscle tissues expressed splitting of muscle fibres and degeneration in muscle bundles. However, sections of tissues collected from farmed fish have normal morphology and no anomalies. The histopathological assessment indicated various cellular, biochemical, and histological changes in response to the contamination in the vicinity of the fish.

## 1. Introduction

The global concern with heavy metals (HMs) in the marine environment, especially in coastal areas, is due to the development of human settlements and the consumption of marine organisms. Because HMs bioaccumulate in tissues and organs, biomagnifying in the upper levels of the trophic web [1,2], they usually represent a direct source of toxic exposure for consumers (predators) and humans [3]. Some studies have reported intoxications from fish consumption in humans, resulting in renal dysfunction, abdominal pain, fatigue, headache, loss of appetite, and memory.

River Chenab is one of the important rivers of Punjab, Pakistan. It is approximately 960 Km long. The untreated sewage and industrial toxic wastes from Faisalabad city are added to the Chenab River through Chakbandi drain. (CMD) Sewage and fertilizers produce algal bloom, which decreases dissolved oxygen in water, ultimately leading to the death of aquatic life [4]. Oxygen declines in water lead to digestive, respiratory, reproductive, and physiological abnormalities in the functions of aquatic life.

Water contaminated by industrial and municipal sewage has also been responsible for developing skin lesions, teratogenic effects, skin ulceration, and fungal disease in fish communicable in humans. Feeding contaminated food leads to bioaccumulation in the food chain [5]. Defective proteins affect the functioning of an organism by altering the physiology of cells [6]. Heavy metals concentrate by binding biological active constituents of lipids, proteins, amino acids, carbohydrates, etc., producing health defects and can be transmitted to the next generation through defective genes [7]. Xenobiotic binds to specific receptors on the cell surface, cytoplasm, or cell organelles and induces cellular processes that have toxic and adverse effects on cells, organs, individuals, and even the whole population [8,9]. Fish are used because it is a bioindicator of water quality because of their sensitivity to water pollution [10,11]. The different fish species indicated the varied response to pollutants with sizes and ages [12].

Compared to other aquatic organisms, these changes are easily observed in fish through their modified endocrine, nervous, osmoregulatory, and immune systems [13,14]. Tilapia is one of the best species to use as model for pollution studies [15]. Besides having good taste, it has excellent nutrition qualities [16]. It is a one of the popular fish due to its high nutritional compared to the freshwater fish species [15]. Tilapia feeds in shallow waters and is sensitive to physiological fluctuation. The small-sized tilapia prefers to eat animal origin food to larger-sized ones selected to feed on phytoplankton, detritus, and macrophytes. It shows variation in eating habits from animal omnivores to herbivores feeding with an increase in age. Such fish can be good research material for consuming various food with surface water feeding habits [17,18]. Histological alterations act as a biomarker of contaminants [19,20] and provide direct and indirect effects on animal tissues [20]. It also indicates the whole population’s health in the ecosystem [21,22,23]. This research has been planned to ascertain the effects of pollution in *Oreochromis niloticus* by determining histological changes in fish muscles, liver, gills, and kidneys and their relative sensitivity level.

## 2. Materials and Methods

### 2.1. Collection of Fish and Water Samples

**Figure 1:**
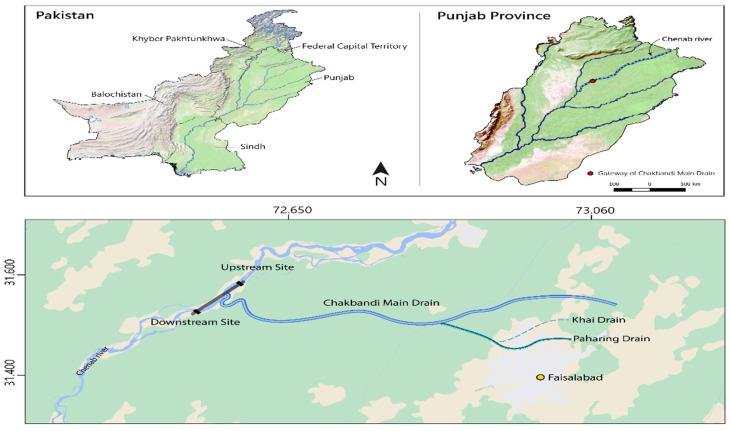
Site map of the study area indicating experimental sites and the gateway of Chakbandi main drain into the River Chenab (Google source).

*Oreochromis niloticus* specimens were collected (n = 7) from the Chenab River upstream and downstream of the Chak Bandi drain (CMD) entrance into the river (Figure 1). Control group specimens were procured from Government Fish Seed Hatchery, Satiana Road, Faisalabad, Pakistan. The specimens of *O. niloticus* were brought to the Research Laboratory, Department of Zoology, Government College University, Faisalabad, for analysis. Water samples were collected in 1.5 L polyethylene water bottles from each site of the fish harvest. They were analyzed fresh for selected water quality parameters [24]. Metals were detected by heavy metal kits (Merck, Germany) and atomic absorption spectrophotometer (2000 series, High-Technologies Corporation, Chiyoda, Tokyo, Japan). Quality assurance and control (QA/QC) procedures were carried out to estimate the studied metals from water. All glassware used for the analyses was acid washed and thoroughly rinsed with deionized water. Reagent blanks were treated as samples and digested simultaneously using the same procedure for percentage recovery of the method, a triplicate of certified reference materials as part of the quality control.

### 2.2. Histopathological Procedure

Fish were dissected to remove liver, muscle, kidney, and gills. Tissues were further processed with three replicates by following the method described by [25].

#### 2.2.1. Fixation

Histological tissues of fish were fixed in sera for 3–6 h. Fixation solution of absolute alcohol, formaldehyde, and glacial acetic acid in a 60 mL:30 mL:10 mL, respectively.

#### 2.2.2. Dehydration

After fixation, tissue slices were kept in tagged baskets. Dehydration was done with 30, 50, 70, 90, and 100 percent ascending grades of alcohol for 2 h. Then tissues were made clear by conveying to a clearing agent (cedar wood oil) at room temperature.

#### 2.2.3. Infiltration and Embedding

Tissues were inserted in molds and paraffin wax for 40–60 min at 59 °C in the oven. Bubbles were erased, and wax blocks were retained in the refrigerator for solidification.

#### 2.2.4. Sectioning

After trimming the wax block with a blade and fixing it on the wooden block. A microtome was used to cut tissues into 2–5 microns’ sections. Adhesive material such as albumin was placed on the slide. After cutting with a microtome, ribbons were moved toward the sticky slide. These slides were kept in the oven overnight at 37 °C.

#### 2.2.5. Staining

Xylene was used for removing wax from the sections. Tissues were hydrated with descending series of alcohol. Hematoxylin and eosin stain were used sequentially. Then the tissues on slides were covered with a coverslip by using Canada balsam.

#### 2.2.6. Examination

Slides were examined under a microscope. Photomicrography was performed under 40× and 60×. Then histopathological variations were observed and compared with control samples.

## 3. Results

Physicochemical parameters indicated that the water quality of the River Chenab was inferior due to the pollution bestowed by tributary wastewater from CMD. Water quality parameters and selected metals analyzed downstream CMD were found enough higher than the permissible limits defined by WHO indicating an acute level of aquatic toxicity (Table 1). Experimental specimen collected downstream gateway of CMD into the River Chenab showed several changes in body organs. These changes were compared with the respective control fish.

**Figure 2:**
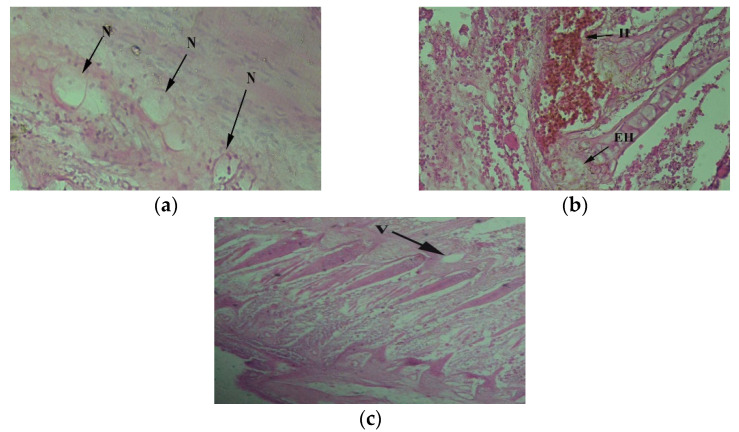
*Oreochromis niloticus* collected downstream of CMD gateway to the Chenab River, indicating defective gill tissue accompanied with (**a**) necrosis (N) 40×, (**b**) hemorrhage (H) and epithelial hyperplasia (EH) 40× and (**c**) vacuolation (V) 40×.

**Figure 3:**
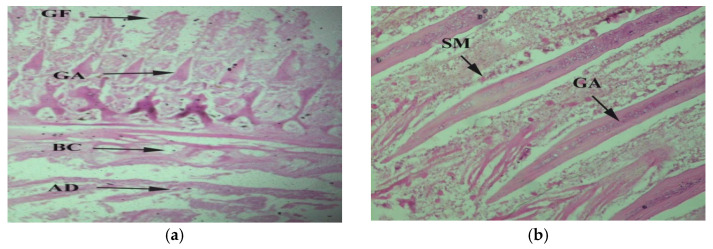
Farmed fish (*Oreochromius niloticus*) acting as control indicating normal gills tissue with (**a**) gill filament (GF), gill arch (GA), branchial capillaries (BC), and adipose tissues (AD) 40×; (**b**) accompanied with skeletal muscles (SK) and gill arch (GA) 40×.

Gills perform several vital functions, such as excretion of metabolic waste, ion regulation, and gas exchange. The gills of tilapia obtained from downstream area fish exhibited necrosis (Figure 2a), hemorrhage, and CMD areas indicated vacuolation (Figure 2c). Structurally, the gills of control fish were composed of primary and secondary lamellae and mucous cells, and chloride cells were less in number or completely absent. Gills architecture indicated primary lamellae having chondrocyte skeleton and thread-like in secondary lamellae, and it comprises pillar cells with a protective covering of mucous cells. Gill filament, gill arch, branchial capillaries, and adipose tissues were also present (Figure 3a). Blood is supplied through the gill arch as illuminated (Figure 3b).

**Figure 4:**
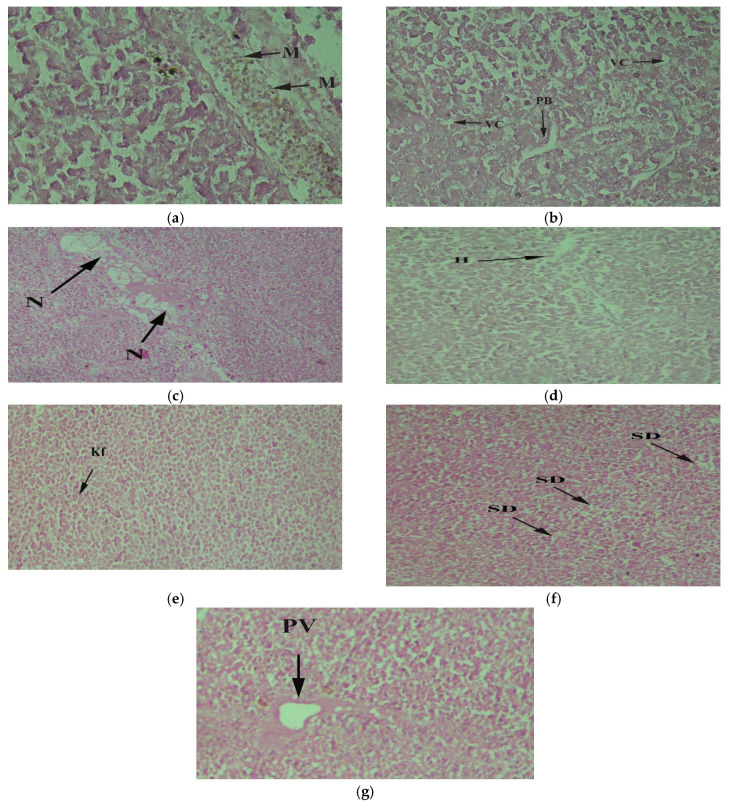
*Oreochromis niloticus* collected downstream of CMD gateway to Chenab River indicating defective liver tissue accompanied with (**a**) vacuolated cytoplasm (VC) (**b**) vacuolated cytoplasm (VC) and bile ductless (PB) proliferation 20×; (**c**) necrosis (N) 40×; (**d**) hypertrophy (H) 20×; Kupffer cell (KF) 40×; (**e**) sinusoid dilation (SD) 20×; (**f**) portal vein (PV) 20×. (**g**) no changes in control fish.

The liver filters the blood and supply throughout the body, and it also plays a vital role in metabolism. Liver tissues of tilapia collected from downstream having vacuolated cytoplasm and found with bile duct proliferation (Figure 4a). Melanomacrophages were also found to be present in large numbers (Figure 4b), while necrosis was also seen (Figure 4c). Tissues of upstream specimens were affected by hypertrophy (Figure 4d) and had more Kupffer cells than expected (Figure 4e). Liver tissues of farmed fish have anastomosed lamina surrounded by sinusoids. Hepatic parenchyma was found in interlobular connective tissues, while hepatocytes are polyhedral cells with a transparent cell membrane. The spherical nucleus was present in the center. Kupffer cells and mitochondria was also present in the lumen of the sinusoid (Figure 4f) in the contaminated fish compared to the control (Figure 4g).

**Figure 5:**
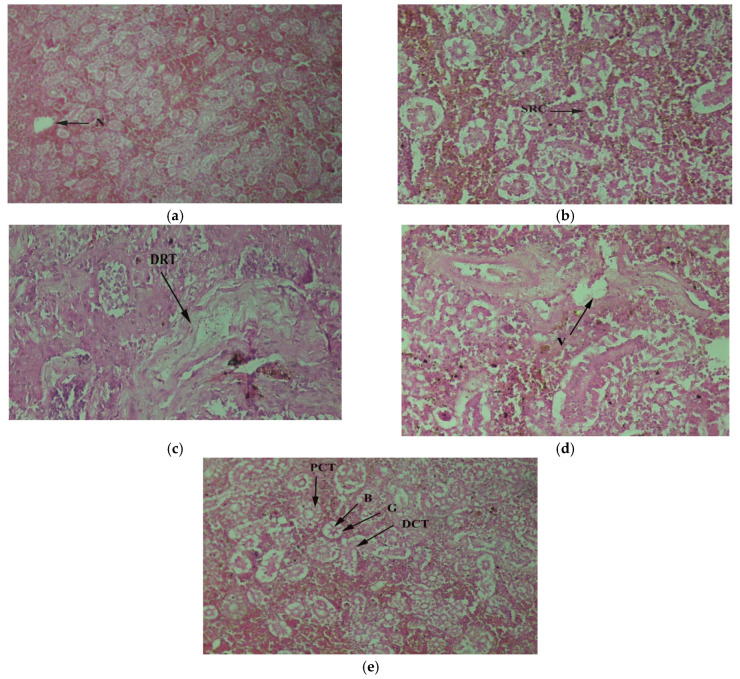
*Oreochromis niloticus* collected downstream of CMD gateway to the Chenab River indicating defective kidney tissue with (**a**) necrosis (N) 20×; (**b**) shrunk renal cortex (SRC) 20×; (**c**) destructive renal tubules (DRT) 40×; (**d**) vacuolation (V) 40×; (**e**) Bowman’s capsule (B), glomerulus (G), distal convoluted tubules (DCT), proximal convoluted tubules (PCT) 40×.

The kidney removes waste products from the body and regulates body fluids. The fish’s kidneys collected from the downstream exhibited necrosis, shrunk renal cortex, and destructive renal tubules (Figure 5a–c). The kidney of tilapia collected from upstream had vacuolation (Figure 5d). The kidney of control specimens showed a normal structure consisting of many renal corpuscles that consisted of normal glomerulus and Bowman’s capsule, and Bowman's capsule was surrounded by epithelial cells (Figure 5e). Proximal tubules were lined by columnar epithelial cells with a brush-like border. Collecting ducts were present everywhere in the kidney, and they were more prominent in diameter and made up of columnar epithelial cells without brush border.

**Figure 6:**
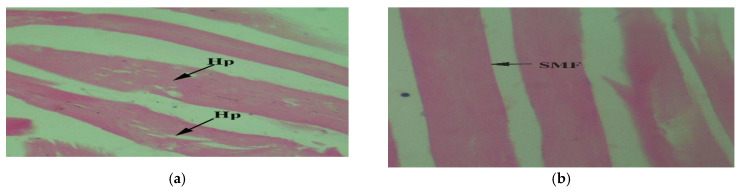

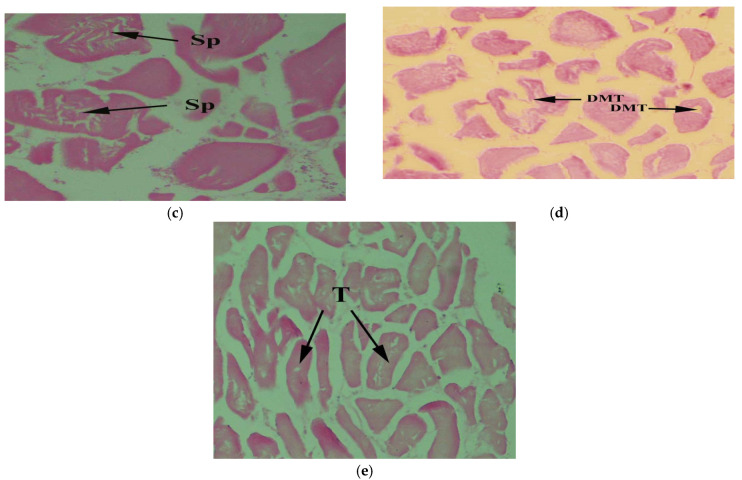
*Oreochromis niloticus* collected downstream of CMD gateway to the Chenab River indicating defective muscle tissue with (**a**) hypertrophy of muscle bundle (Hp) 40×; (**b**) swollen muscle fibres (SMF) 40×; (**c**) the splitting of muscle fibers (Sp) 40×; (**d**) degeneration in muscle tissues (DMT) 40×; (**e**) Farmed fish (*Oreochromis niloticus*) acting as control indicating normal transversely cut muscle tissues (T) 40×.

Muscles provide facilitation in movement. Transversely cut muscle tissues in tilapia collected downstream areas showed hypertrophy (Figure 6a) and swollen myofibers (Figure 6b). Upstream specimens were found to be affected by splitting muscle fibers (Figure 6c) and degeneration in the muscle bundle (Figure 6d). Muscle fibers of control tilapia were multinucleated. Nuclei were present under the sarcolemma, and cells were sheeted by sarcolemma. Cells were found with several fibers that were made up of many myofibrils. Loose connective tissues filled the spaces between myocytes. In rhabdomyocytes, alternating light and dark bands were present (Figure 6e).

## 4. Discussion

Water quality can be determined through physical, chemical, and biological factors. Heavy metal accumulations in the tissues of aquatic animals produce cytotoxicity [14,26] and have serious health issues due to their persistent nature [27]. Present findings of metals also indicated acute aquatic toxicity due to the higher concentrations of metals in the water samples. These findings agree with the previous studies [12,26,28]. Heavy metals are biomagnified in the food chain through polluted food sources of fish [28,29]. Pollution affects fish organs directly. Monitoring the level of heavy metals in the water alone is not enough in the communities in an ecosystem [26]. The most prominent basic harm coming about from the heavy metals (HM) may be in vital organs in the fish. As a result of the increased concentration of HM, the histological structure may alter, and physiological stretch may happen. This stretch can cause a few changes in metabolic functions at the cellular level and in tissues. In this context, histological examinations of examples to decide the contamination of water biological systems can give valuable data on the well-being of that ecosystem [29].

The present study observed gills necrosis, hemorrhage, epithelial hyperplasia, and vacuolization. Parallelism in results was seen by [30]. They pointed out gills’ morphological alternation under chemical stress [31,32]. They reported that the gills lamellar degradation and disorganization in different species collected from three various sites in the Karasu River of Turkey. Coherence in results was observed by [33] in the instance of *Oreochromis niloticus*. They pointed out hyperplasia, edema, and proliferation of filamentary epithelium due to sewage water in densely populated areas of Portugal. They concluded that the necrosis might be caused due to a low oxygen level.

In present studies, the liver tissues of tilapia indicated hypertrophy, frequent Kupffer cells, bile duct proliferation, melanomacrophages, necrosis, and vacuolated cytoplasm. Akin abnormalities were reported by [34] in a matter of *Tilapia zillii*. Biological disarrangement, hypertrophy, and melanomacrophages were also observed in the fish treated with different aluminum concentrations by [34,35]. They emphasized that vacuolization of hepatocytes, degenerative hepatocytes, necrosis, and disruption of typical architecture was produced downstream of Sutlej River due to Buddha Nullah pollution at Ludhiana. Necrosis found in the centrilobular zone might be due to a high level of toxic chemicals, leading to hepatocytes loss. Similar results have been demonstrated by [36] with *Squalius cephalus*. They pointed out that melanomacrophages, hypertrophy, and necrosis were produced due to polluted water with several metals in the Karasu River of Turkey, corroborating this project’s findings. Mitochondrial granules, hepatocytes, and frequent Kupffer cells might result from some lethal heavy metals.

In this study, the kidney tissues of tilapia showed destructive renal tubules, shrunk renal cortex, vacuolation, necrosis, and dilation of renal tubules. Similar results were also reported by [37] on *Cyprinus carpio*. Ref. [38] carried out a previous study in the case of perch and *Lates calcarifer*. They pointed out variation in tubules and glomerulus by exposure to cadmium. Ref. [39] mentioned the necrotic changes in the focal area and renal tubules, edema of Bowman's capsule, hemorrhage, and vacuolation induced due to polluted water. Ref. [25] highlighted necrosis of tubular epithelium, hypertrophy of renal tubules, narrowing of tubule lumen, glomerulus constriction, and Bowman's capsule space elevation was created in the case of *Cirrhinus mrigala* due to fenvalerate.

Histopathology of muscle tissue showed splitting muscle fibers, degeneration, hypertrophy in the muscle bundle, and swollen myofibers. Some anomalies such as edema, thickening, and detachment of muscles were narrated by [40] when *Lates calcarifer* was exposed to copper. Splitting of muscle fibres might be due to hypoxic conditions. Such anomalies were also reported by [41] in the event of Nail tilapia. They highlighted that oedema, in muscle fibers, fatty tissue, necrosis, splitting of muscle fibers, necrosis, and infiltration of lymphocytes induced due to polluted water in the Edku Lake. Variation in results might be due to a complex discharge mixture of different sites. These abnormalities were observed by [42,43] in the *Mugil cephalus*; as a loss and splitting off muscle fibers, swelling in muscle fibers, and breakdown of the muscle bundle induced by heavy metals in east Barbic-Correntyne, Guyana. In muscle tissue, edema occurs due to fluid seepage, which generates muscle inflammation. A similar study was conducted on *Labeo rohita* by [1,2], who reported that shortening of muscle bundles, necrosis, and edema was produced due to heavy metals.

## 5. Conclusions

It has been concluded that heavy metals have deleterious effects on vital organs of *Oreochromis niloticus*. This fish species showed significant irreversible tissue damage in the liver, muscle, kidney, and gills in response to pollution. It has been observed that alterations in histopathogical changes was strongly related with the level of pollution. This study could be used as an alarming warning of pollution load that creates constant stress conditions for aquatic life, notably fish species inhabitant of the River Chenab. Thus, taking necessary actions to improve the ecological status of the respective water bodies must not solely focus on water quality but include steps to deal with the contamination of sediments, in which bioactive and persistent substances could have been accumulating for decades, to ensure a sustainable improvement of aquatic ecosystems in densely populated areas of industrialised and developing countries. There is a dire need for the Ministry of Public Health to ensure strict implementation of environmental laws so that future human generations enjoy good health.

## Figures and Tables

**Table 1:** Water quality parameters of the River Chenab due to untreated wastes disposal by CMD.

Water Quality Parameters (mg/L)	Downstream Gateway of CMD	Upstream Gateway of CMD	Permissible Limits
Lead	2.975 ± 0.06 c	0.15 ± 0.02 a	D: 0.05 mg/L, P: **
Copper	2.002 ± 0.07 a	0.84 ± 0.08 b	D: 0.05 mg/L, P: 1.5 mg/L
Cadmium	0.26 ± 0.02 a	0.022 ± 0.0 b	D: 0.01 mg/L, P: **
Mercury	1.445 ± 0.03 b	0.011 ± 0.0 c	D: 0.001 mg/L, P: **
Cobalt	0.979 ± 0.003 b	0.833 ± 0.05 a	D: 0.05 mg/L
Tin	0.488 ± 0.01 a	0.019 ± 0.0 b	D: 0.01 mg/L, P: **
Chromium	0.676 ± 0.04 a	0.28 ± 0.05 b	D: 0.05 mg/L, P: **
Zinc	0.54 ± 0.03 c	0.19 ± 0.03 b	D: 5 mg/L, P: 15 mg/L
Manganese	2.73 ± 0.1 b	1.81± 0.06 a	D: 0.1 mg/L, P: 0.3 mg/L
Sulphates	442 ± 5.71 a	346± 6.93 c	D: <400 mg/L, P: 400 mg/L
Salinity	1909 ± 7.68 a	412 ± 3.02 b	P: <100 mg/L
Phenols	2.31 ± 0.03 b	0.68 ± 0.04 c	D: 0.001 mg/L, P: 0.002 mg/L
Conductivity (µS/cm)	3.31 ± 0.02 b	1.29 ± 0.07 a	D: 2.1 µS/cm, P: 2.1 µS/cm
Hardness	576.19 ± 8.12 c	207.42 ± 4.42 b	P: 120–170 mg/L P: **
BOD	76.94 ± 0.39 c	43.7± 0.07 a	† D: 30 mg/L, P: **
COD	193.95 ± 1.93 c	70.4 ± 0.22 a	† D: 250 mg/L, P: **
TSS	308 ± 2.44 a	209 ± 5.21 c	D: 100mg/L, P: **
TDS	2411 ± 6.87 a	1318 ± 8.01 b	D: 500 mg/L, P: 2000 mg/L
pH	11.8 ± 0.25 a	8.0 ± 0.07 b	D: 6.5–8.5, P: **

Values (Mean ± SE) are averages. CMD: Chakbandi Main Drain, BOD: Biological oxygen demand, COD: Chemical oxygen demand. TDS: Total Dissolved solids, TSS: Total suspended solids. D; Desirable limits. P; Permissible limits. †; Effluent inland surface water quality standards. ** No relaxation. Different letters in a row indicates a statistically significant difference (*p* < 0.05).

## Data Availability

Available on request from first author (Sana Shahid).

## References

[1] Thabet I.A., Tawadrous W.E., Samy A.M. (2019). Pollution induced change of liver of *Oreochromis niloticus*: Metals accumulation and histopathological response. World J. Adv. Res. Rev..

[2] Savassi L.A., Paschoalini A.L., Arantes F.P., Rizzo E., Bazzoli N. (2020). Heavy metal contamination in a highly consumed Brazilian fish: Immunohistochemical and histopathological assessments. Environ. Monit. Assess..

[3] Brázová T., Šalamún P., Miklisová D., Šestinová O., Findoráková L., Hanzelová V., Oros M. (2021). Transfer of Heavy Metals Through Three Components: Sediments, Plants and Fish in the Area with Previous Mining Activity. Bull. Environ. Contam. Toxicol..

[4] Rodhe W. (1969). Crystallization of eutrophication concepts in North Europe. Eutrophication, Causes, Consequences.

[5] Kim H.K.E., Kabir S., Jahan A. (2016). A review on the distribution of Hg in the environment and its humanhealth impacts. J. Hazard. Mater..

[6] Baig J.A., Kazi T.G., Arain M.B., Afridi H.I., Kandhro G.A., Sarfraz R.A., Jamal M.K., Shah A.Q. (2009). Evaluation of arsenic and other physico-chemical parameters of surface and ground water of Jamshoro, Pakistan. J. Hazard. Mater..

[7] Lee S., Kopp F., Chang T.C., Sataluri A., Chen B., Sivakumar S., Yu H., Xie Y., Mendell J.T. (2016). Noncoding RNA NORAD regulates genomic stability by sequestering PUMILIO proteins. Cell.

[8] Fent K., Woodin B.R., Stegeman J.J. (1998). Effects of triphenyltin and other organotins on hepatic monooxygenase system in fish. Comp. Biochem. Physiol. Part C Pharmacol. Toxicol. Endocrinol..

[9] Rogiers V., Vercruysse A. (1998). Hepatocyte cultures in drug metabolism and toxicological research and testing. Cytochrome P450 Protocols.

[10] Das S.K., Chakrabarty D. (2007). The use of fish community structure as a measure of ecological degradation: A case study in two tropical rivers of India. Biosystems.

[11] Mishra R., Shukla S.P. (2003). Endosulfan effects on muscle malate dehydrogenase of the freshwater catfish *Clarias batrachus*. Ecotoxicol. Environ. Saf..

[12] Burger J., Gaines K.F., Boring C.S., Stephens W.L., Snodgrass J., Dixon C., Mcmahon M., Shukla S., Shukla T., Gochfeld M. (2002). Metal levels in fish from the Savannah River: Potential hazards to fish and other receptors. Environ. Res..

[13] Song Y., Salbu B., Heier L.S., Teien H.C., Lind O.C., Oughton D., Petersen D.K., Rosseland B.O., Skipperud L., Tollefsen K.E. (2012). Early stress responses in Atlantic salmon (*Salmo salar*) exposed to environmentally relevant concentrations of uranium. Aquat. Toxicol..

[14] Terra B.F., Araújo F.G., Calza C.F., Lopes R.T., Teixeira T.P. (2007). Heavy metal in tissues of three fish species from different trophic levels in a tropical Brazilian river. Water Air Soil Pollut..

[15] Fitzsimmons K., Martinez-Garcia R., Gonzalez-Alanis P. Why tilapia is becoming the most important food fish on the planet. Better science, better fish, better life. In Proceedings of the 9th International Symposium on Tilapia in Aquaculture, Shanghai Ocean University, Shanghai, China, 21–24 April 2011.

[16] Hernandez-Sanchez F., Aguilera-Morales M.E. (2012). Nutritional richness and importance of the consumption of tilapia in the Papaloapan Region. REDVET Rev. Elec. Vet..

[17] Mjoun K., Rosentrater K., Brown M.L. (2019). Tilapia: Profile and Economic Importance. http://openprairie.sdstate.edu/extensionfact/163.

[18] Greenfield B.K., Teh S.J., Ross J.R.M., Hunt J., Zhang G., Davis J.A., Ichikawa G., Crane D., Hung S.S.O., Deng D. (2008). Contaminant concentrations and histopathological effects in *Sacramento splittail* (*Pogonichthys macrolepidotus*). Arch. Environ. Contam. Toxicol..

[19] (2019). Wikipedia. https://en.m.wikipedia.org/wiki/Catfish.

[20] Paithane K.T., Sonawane DLBandare R.Y., More P.R. (2012). Histopathological changes due to induced dimethoate in the liver of freshwater fish Channa punctatus from River Shivana, Aurangabad (ms) India. Inter. Q. J. Environ. Sci..

[21] Costa P.M., Diniz M.S., Caeiro S., Lobo J., Martins M., Ferreira A.M., Caetano M., Calos T.A.D., Costa M.H. (2009). Histological biomarkers in liver and gills of juvenile *Solea senegalensis* exposed to contaminated estuarine sediments: A weighted indices approach. Aquat. Toxicol..

[22] Schwaiger J., Ferling H., Mallow U., Wintermayr H., Negele R.D. (2004). Toxic effects of the non-steroidal anti-inflammatory drug diclofenac: Part I: Histopathological alterations and bioaccumulation in rainbow trout. Aquat. Toxicol..

[23] Wester P.W., Canton J.H. (1991). The usefulness of histopathology in aquatic toxicity studies. Comp. Biochem. Physiol. C Comp. Pharmaol. Toxicol..

[24] Boyd C.E. (1981). Water Quality in Warm Water Fish Ponds.

[25] Sultana T., Butt K., Sultana S., Al-Ghanim K.A., Mubashra R., Bashir N., Ahmed Z., Ashraf A., Mahboob S. (2016). Histopathological changes in liver, gills and intestine of *Labeo rohita* inhabiting industrial waste contaminated water of River Ravi. Pak. J. Zool..

[26] Yildirim Y., Gonulalan Z., Narin I., Soylak M. (2019). Evaluation of trace heavy metal levels of some fish species sold at retail in Kayseri, Turkey. Environ. Monit. Asses..

[27] Jaishankar M., Seten T.T., Anbalagan N., Mathew B., Beeregowda K.N. (2014). Toxicity, mechanism and health effects of some heavy metals. Interdiscip. Toxicol..

[28] Malik N., Biswas A.K., Qureshi T.A., Borana K., Virha R. (2014). Bioaccumulation of heavy metals in fish tissues of a freshwater lake of Bhopal. Environ. Monit. Asses..

[29] Javed M. (2004). comparison of selected heavy metals toxicity in the planktonic biota of the river Ravi. Indian J. Biol. Sci..

[30] Yancheva V., Velcheva I., Stoyanova S., Georgieva E. (2016). Histological biomarkers in fish as a tool in ecological risk assessment and monitoring programs: A review. Appl. Ecol. Environ. Res..

[31] Ramudu K.R., Dash G. (2015). Histopathological alterations in the vital organs of Indian Major Carps with parasitic infestation in fish farms West Bengal, India. Drug Dev. Ther..

[32] Mallatt J. (1985). Fish gill structural changes induced by toxicants and other irritants: A statistical review. Can. J. Fish Aquat. Sci..

[33] Fernandes C., Fontainhas-Fernandes A., Monteiro S.M., Salgado M.A. (2007). Histopathological gill changes in wild leaping grey mullet (*Liza saliens*) from the Esmoriz-Paramos coastal lagoon, Portugal. Environ. Toxicol..

[34] Hadi A.A., Alwan S.F. (2012). Histopathological changes in gills, liver and kidney of fresh water fish, *Tilapia zillii*, exposed to aluminum. Int. Pharm. Life Sci..

[35] Jaidka A., Hundal S.S. (2016). Histological changes in gills and liver of fishes in River Sutlej as an effect of Buddha Nullah pollution at Ludhian. Int. J. Life Sci..

[36] Kaur S., Khera K.S., Kondal J.K. (2018). Heavy metal induced histopathological alterations in liver, muscle and kidney of freshwater cyprinid, *Labeo rohita* (Hamilton). J. Entomol. Zool. Stud..

[37] Dane H., Sisman T.A. (2017). Histopathological study on the freshwater fish species chub (*Squalius cephalus*) in the Karasu River, Turkey. Turk. J. Zool..

[38] Tilak K.S., Rao D.K., Veeraiah K. (2005). Effects of chlorpyrifos on histopathology of the fish *Catla catl*. J. Ecotoxicol. Environ. Monit..

[39] Thophon S., Kruatrachue M., Upatham E.S., Pokethitiyook P., Sahaphong S., Jaritkhuan S. (2003). Histopathological alterations of white seabass, *Lates calcarifer*, in acute and subchronic cadmium exposure. Environ. Pollut..

[40] Yokote M., Hibiya T. (1982). Digestive system. An Atlas of Histology—Normal and Pathological Features Commercial Deltamethrin on the Structure of the Gill.

[41] Maharajan A., Kitto M.R., Paruruckumani P.S., Ganapiriya V. (2016). Histopathology biomarker responses in Asian sea bass, Lates calcarifer (Bloch) exposed to Copper. J. Basic Appl. Zool..

[42] Wassif E., Mourad M.H., Haredi A.M.M., Tanekhy M. (2017). Biochemical and Histopathological Changes in Nile Tilapia, *Oreochromis niloticus* at Lake Edku. Alex. J. Vet. Sci..

[43] Rajeshkumar S., Karunamurthy D., Halley G., Munuswamy N. (2015). An integrated use of histological and ultra-structural biomarkers in *Mugil cephalus* for assessing heavy metal pollution in east Berbice-Corentyne, Guyana. Int. J. Bioassays.

